# Extracts and Gleanings

**Published:** 1871-01

**Authors:** 


### PART II.

### Abridged Extracts and Gleanings from our Exchanges

*MULTUM IN PARVO.*

---

### REMARKS ON THE TREATMENT OF ASTHMA.

Dr. Hyde Salter, the greatest living authority upon Asthma, says (in the London Lancet, Jan. 30th,) he has used *Belladonna* extensively for the last two years in this disease with the best success. He directs the patient to take ten drops of the tincture of *belladonna* every night on going to bed—to begin at ten minims, and gradually to increase the dose until the head and sight become affected. Dr. Salter believes belladonna has not been given in large enough doses—it should produce its physiological effects, otherwise we have no right to say it has been fairly tried. He thinks to give ten minim doses three times a day is simply worthless. The advantages he claims for his method of using belladonna are as follows :

1. Given at night, it meets an anticipated attack, which usually occurs at that time.

2. By gradually increasing the dose, the proper effect is had without fear.

3. In those cases in which Asthma yields before the head or sight is appreciably affected, it enables the administrator to stop short as soon as relief is obtained, and thus spares the patient any of the disagreeable effects of the drug.

4. By giving it only once in twenty-four hours, a larger dose may be given.

5. By confining the dose to bed-time, the days are passed in comfort.

6. The proper dose for each individual is ascertained—a very important point. Some patients cannot take twenty minims without unpleasant effects ; others are able to take a drachm three times in the same interval, without any inconvenience.

7. It is prophylactic, given at night. It does more than spare the patient an attack ; it breaks *pro tanto* that chain of sequences which is the very life of the morbid tendency.

While belladonna is certainly a very valuable remedy in spasmodic Asthma, it should, however, in our opinion, not be solely relied upon in other forms of the disease—nor indeed in the purely spasmodic variety. Dr. J. P. Whittemore, in the *Cincinnati Lancet & Observer*, December 1870, gives, in many particulars, our views in regard to the treatment of Asthma. He relies mainly upon the iodide of potassium. He says, "he has used this drug for twenty years; that it never fails to give relief." He gives five grains at bed-time, but does not repeat the dose unless the attack is also repeated, which he thinks is the secret of his success with the remedy. While it is more valuable in the bronchial form, he thinks it is useful in the other varieties also. Dr. H. T. Broadwell advises the following method for the radical cure of the disease, which we recommend. He says: "Especial attention should be given to bathing the body every day with cold water, beginning with it at first tepid, and then using it cooler and cooler, until it can be used cold." Salt or Saleratus may be added to the water, which should be done on rising in the morning. After bathing, the skin should be thoroughly dried, by brisk friction, with a coarse towel. This prevents the patient taking cold at every atmospherical change. At the same time he "earnestly recommends" the following prescription:

| | |
|---|---|
| ℞—Tr. Cinchona, comp. . . . | ℥ ss. |
| Tr. Gentian, comp. . . . | ℥ ii. |
| Ext. Stillingia, comp. . . . | ~~ʒ i.~~ |
| Potass. Iodide, . . . | ~~℥ iss.~~ |
| Aquæ, . . . | ℥ ss. |

Potass. Iodide, in aquæ, solve, Misce et

Sig—A table-spoonful three times a day before eating.

This prescription has proven entirely satisfactory, in the hands of the Senior editor, after the failure of belladonna. His experience corroborates that of Dr. Broadwell, when he says, "it is wonderful in its results" in that form of the disease where bronchitis is combined with Asthma.

---

### TREATMENT OF PULMONARY CONSUMPTION.

Dr. C. J. B. Williams, of London, sums up, in the *Lancet*, the result of his forty years of observation on this subject somewhat as follows:

When he began practice two years was regarded as the limit of life of the consumptive.

Ten years showed a marked improvement in results of treatment, mainly from the use of alterative tonics, such as iodide of potassium, with sarsaparilla, or other vegetable tonics.

At this time a good article of cod liver oil began to be procurable, and he has no hesitancy in saying that this agent has done more for the consumptive than all other means put together. Its usefulness and efficiency have gone on increasing in proportion to the greater facility for obtaining it pure, and to the improvement in the manner of giving it in combination with various tonics, and in conjunction with certain rules of diet and regimen.

As a result, during the forty years, the average life of the consumptive under proper treatment, has increased from two to eight years.

Regarding consumption as a disease of degeneration and decay, his treatment is, of course, for the most part sustaining and invigorating, consisting of such means as rich food, tonics, stimulants, fresh air and the like. But as many cases originate in inflammation, and are aggravated and kept up by it, a mild antiphlogistic treatment is at certain stages often called for, such as salines, at times antimony, cataplasms, leeches and blisters. He thinks there is at present a tendency to ignore too much this indication. The tonics he makes use of mainly are quinine, salacine and iron; but the pure bitter tonics are often found very beneficial, as also mineral acids, which he almost invariably gives in conjunction with the cod liver oil.

But the latter agent is most of all important.

This he rarely gives in doses greater than a half-ounce three times a day, and he interdicts, while it is being administered, all other fatty substances.

When cod liver oil cannot be taken, he falls back upon the old treatment, with iodide of potassium, nitric acid and a bitter tonic, adding to each dose a drachm or two of pure glycerine.

The hypophosphites of soda and lime have in his hands proved decidedly beneficial in certain cases.

In cases where the larynx or trachea are affected, or there is convulsive cough, or offensive expectoration, inhalations are quite valuable.

He regards the inhaling instruments of little use, but prefers a quart jug of hot water into which the medicine is dropped. A napkin rolled into the shape of a tube is placed over the nozzle, and from this the patient inhales the steam impregnated with the medicine. This to be used a few minutes each night.

The drugs proven to be most valuable for this means of exhibition are creosote or carbolic acid, iodine, chloroform, oil of turpentine, and juice or extract of hemlock; a few drops of one or each of several being dropped into the water when used.

Under careful treatment the life of the consumptive may be prolonged for many years, and in not a few cases he may be cured.—*Richmond and Louisville Medical Journal.*

---

SCARLET FEVER.—Dr. Peter Hood (British Medical Journal, Feb. 6, 1869,) details his treatment of *Scarlet Fever.* He says: when the stomach has been relieved by emesis, and the liver and bowels acted upon by an efficient dose of medicine, he then avails himself of the antiseptic property of quinine, which drug, he believes, is as powerful in destroying the Scarlatinal germ as it is potent in the cure of ague. He says no one will fail to employ the remedy after once witnessing the progress of such cases twenty-four hours afterwards. In the worst forms, he combines iron with the quinine. Dr. Thompson (Lancet Feb. 27,) has, for fifteen years, successfully adopted, in *Scarlatina*, the use of warm baths, repeated as often as the strength of the patient will permit, or severity of the attack allow. During convalesence, one bath may be used every day or every other day—its temperature being gradually reduced. Under his method, the terrible sequelæ of the disease is seldom met with.

The Senior editor of this Journal has found great advantage in administering, in Scarlet Fever, Cream of Tartar. He gives about an ounce to a quart of warm water, acidulated with lemon juice, to be drank *ad libitum*, using the warm bath, as the case may require. He believes the Scarlatina poison requires a definite time for elimination, and that by this treatment elimination takes place rapidly, and the patient rescued, in the larger number of instances, from danger.

STRYCHNIA IN INTERMITTENT FEVER.–This alkaloid has been employed with the most marked and happy effects, in many cases of Intermittent Fever, in which quinine and other tonics had been used without success. We have never used Strychnia as an anti-periodic, but as a remedial agent, we are satisfied that its powers are as yet only known and appreciated when given in minute doses, suited to the susceptibility of the subject. It most usually imparts vigor and force to the muscular organization—hence we use it with great benefit in almost any atonic condition of the organization occurring from various causes, and under different circumstances. In atonic dyspepsia, and in that deplorable disease known as Spermatorrhœa, the following prescription is not a specific, but we have yet to learn one that is better :

℞—Sulph. Strychnine, . . gr. ii.
Dilute Phosphoric Acid, . . ℥ iss.

Dissolve the Strychnine completely in the acid. Dose 5 drops in a little water, just before or after each meal.

Bromide of Ammonium in Menorrhagia.—Dr. W. W. Ogden, (*Dominion Med. Journal,*) extols the value of *bromide of ammonium* in "the excessive discharge of blood from the uterus at or about the menstrual period, continuing longer than four or five days." He places the patient on a hair or straw mattress, with light covering, in an easy recumbent position—gives cooling drinks and a mild laxative. After the bowels are moved he gives the bromide at once in doses of 20, 30 or 40 grains, according to the urgency of the case, every three hours, until three doses have been taken; then he reduces the dose to one-half, to be continued as long as required, not neglecting such measures as are calculated to remove the cause.

Nitrate of Silver in Orchitis.—Mr. Smith (British Med. Jour.) uses a strong solution of *Nitrate of Silver* in *Orchitis*. For the first few days, he uses evaporating lotions and gives a purge; he then applies the solution of luna caustic ʒ i. to the ℥ i of distilled water, to the surface of the scrotum, over the inflamed testicle. It usually affords relief in twenty-four hours. He has generally found one application sufficient, but if there should be a slight return of the pain, he uses the solution again. Every case has ended most satisfactorily.

Chloroform as a Febrifuge and Anti-Periodic.—It is said by some to possess these properties but little inferior to Quinine and Arsenic, given in divided doses some hours before the time of access. It has also been used with great success in cases of congestive chills. Dose: Five drops until reaction is fully established. Quinine, in all cases, should be given during the remission.

Remedies for Gonorrhœa.—The oil of the *yellow sandalwood* has been employed with great success in *Gonorrhœa*, by Dr. H. W. A. Beach, (Boston Med. & Surg. Jour.) The white is not efficacious. He uses it also successfully in *Gleet*. He reports over one hundred cases. The best and easiest method of administration is to drop the oil on sugar; for an ordinary case, ten drops three times daily, and for gleet fifteen drops. He has given twenty drops three times daily, without any bad effects. Dr. Perryman (in the Am. Jour. of Med. Sciences,) has employed the

*oil of erigeron*, in ten drop doses, with good results in Gonorrhœa, A medical friend of ours uses the *oil of sassafras* with much advantage. We would suggest a combination of these oils with those of copabia and cubebs, with alcohol and mucilage of gum accaciæ. Drs. Anderson, of Wilmington, N. C. and D. L. Phares, of Mississippi, extol the virtues of tinct. gelseminum in this disease.

An Old but Valuable Remedy for Chronic Inflammation of the Bladder.—Inject four grains of nitrate of silver, dissolved in four ounces of water, into the bladder, and retain for fifteen minutes. This may be repeated every four to eight days, and the strength increased to four gr.—to ounce, if necessary. We have occasionally witnessed the valuable and permanent advantages derived from this method of treating this troublesome and obstinate disease—particularly if pus globules were discovered in the urine, under microscopic examination.

Perchloride of Iron in Post-partum Hemorrhage.—Mr. Norris' experience (British Med. Jour.) in the use of the *perchloride of iron* in *post-partum hemorrhage*, has led him to the following conclusions: 1st. We possess no tropical styptic in efficacy, at all approaching this drug—its effects being certain, perfect and instantaneous. 2d. In post-partum hemorrhage a solution of this salt applied in the form of an intra-uterine injection, is of the utmost value, both in immediately arresting the flow of blood, and in causing a permanent contraction of the uteri. 3d. Its presence in the uterus is not injurious—is anti-septic and beneficial in more ways than one.

Some obstetricians prefer saturating a sponge, and swabbing the interior of the uterus, to using an injection of the solution.

Bromide of Potassium in Puerperal Convulsions.—Dr. T. N. Simmons (Med. Press and Circular,) gives, in *Puerperal Convulsions*, Bromide of Potassium and Antimony. Forty grains of the former, with half a grain of the latter, was given in one case every hour and a half or two hours, until three grains of the antimony were taken. Convalesence was rapid.

Hypophosphites in Tooth-Ache of Pregnancy.—Dr. Sterling, of Burlington. New Jersey, has used successfully the *hypophosphites* in the *Toothache of Pregnancy*, under the belief that the fœtus *in utero* drew from the mother, for its nutrition, the elements of bone and nerve-forming tissues necessary for the maintenance of her osseous, and nervous structures. The theory is plausible, and the practice seems to have been successful.

Carbolic Acid in the Sickness of Pregnancy.—Carbolic Acid is the only remedy which Mr. Garraway, (British Medical Journal,) has ever found of any avail in the *sickness of pregnancy.* He gives drop-doses of the crystal, liquefied by heat, and diffused in half an ounce of thin mucilage, three times a day.

Ergot in Retention of Urine.—Many cases, illustrative of the good effects of Ergot in retention of urine, have been reported. After the usual remedies had failed, when the retention was caused from paralysis of the bladder by over-distension, or paralysis following apoplexy, or vessical atony after parturition, ergot in such cases is valuable, and relieves retention by encouraging the vessical retraction.

Liquor Potassæ in Stranguary.—This is a simple, but is said to be a very efficient remedy in stranguary from blistering with cantharides, in doses of thirty drops every hour. It has also been used with great success in irritation of the bladder, in other cases and from other causes.

Carbolic Acid in Snake-Bite.—Dr. J. W. Hood (Quarterly Journal of Science,) writes that Dr. Boyd has successfully treated two cases of *snake-bite* with carbolic acid—taken internally, and applied as a caustic to the wound. The effect is magical.

Antidote for Carbolic Acid Poisoning.—Messrs. Calvert (Medical Times & Gazette,) wish to make known the fact that sweet or castor oil, in large quantity, is the best antidote to carbolic acid, when swallowed in poisonous doses.

Digitalis in Consumption.—The tincture of digitalis as a means of curing Phthisis, has been used and highly recommended, ever and anon, since 1799. The dose is twenty drops the first day, adding ten drops every day, to be continued 25 to 30 days. We have never used it—have but little faith—but would advise at least a partial trial of the remedy.

Hydrate of Choral in Cerebro-Spinal Meningitis.—Dr. A. Patton (Indiana Med. Journal,) gives four cases of *Cerebro-Spinal Meningitis*, treated with Hydrate of Choral. He says, for the first four days, he used the choral in doses from ten to forty grains every two hours. The uniform effect was to produce complete relaxation of the muscles involved. Delirium and restlessness gave place to quiet and refreshing sleep. Pain, nausea and vomiting were relieved. The treatment of the secondary fever was aconite and iodide of potassium. All his cases were little girls, and remarks "that three of them were saved by this remedy I feel certain."

Chesnut Leaves in Whooping-Cough.—Dr. Ludlow extols (in the Cincinnati Lancet & Observer,) the use of Chesnut leaves in *Whooping-Cough.* He makes an infusion of the leaves, by taking one-half of an ounce of them to the pint of boiling water, and afterwards adds a pint of cold water, to which is added sufficient white sugar to make it palatable. He directs of this, cold, as much as he can get the patient to drink, day and night.

Sulphur in Diptheritic Angina.—Dr. Feyreigne, of Toulouse esteems Sulphur in *diptheritic angina* one of our most valuable remedies. He gives the details of the case of a girl in a dying condition, when the flour of sulphur brought her back to life. The dose was five grammes (about ʒ i) to one glass of water; a teaspoonful of the mixture to be given every hour.

Tannic Acid in Fissures of Anus.—One part of tannic acid dissolved in sixteen parts of glycerine, has succeeded in M. Van Holsbek's hands, in healing *fissures of the anus*, when operations failed. A piece of lint is dipped in the solution, and introduced night and morning into the rectum. The bowels should be kept open.

Creosote in Mercurial Salivation.—This is a very old but very efficient remedy: Creosote ʒ ss; Sage Tea, one pint, to be used as a gargle every hour, for one or two days. Keep the bowels soluble by the use of saline aperionts.

Remedy for Sleeplessness.—*Oxide of Zinc* is said to be a valuable remedy in sleeplessness or hallucination, or trembling, or any other of the disordered nervous symptoms of persons suffering from chronic alcoholism.

Remedy for Ring-Worm.—The *L'Union Medicale* contains the following formula for *ring-worm.* ℞—Washed Sulpher, grs. 22; Carb. Potash, grs. 8; Lard ℥ i.—mix. Continue the application sometime after the apparent cure, in order to prevent a return.

Death from Chloroform.—Dr. Hughes Bennett stated, at the last meeting of the British Association that "he knew of one very sad case that had happened in Edinburgh. A young and beautiful lady, daughter of a barrister, in perfect health, went to a dentist's house one morning, and had a tooth extracted. Five minutes afterwards she was dead. That was only one of many similar cases that had occurred, but had never been published.—*Bost. Med. Jour. Oct.* 1870.

Prescription for Epilepsy.—Dr. Brown—Sequard—states that his usual prescription for Epilepsy is as follows :

℞—Iodide of Potassium, . . . ʒ i.

Bromide of potassium, . . . ℥ i.

Bromide of Ammonium, . . . ʒ iiss.

Bicarbonate of Potash, . . . ℈ ii.

Infusion of Calumba, . . . fl. ℥ vi. mix.

S. A teaspoonful of the mixture to be taken before each meal, and three teaspoonsful at bed-time, with a little water.

In Syphilitic cases, he increases the iodide of potassium. The medicine should be pushed until anæsthesia of the fauces is produced, and an acme-like eruption appears on the face, neck, shoulders, &c. The bromides should be continued fifteen or sixteen months after the attacks have ceased. An occasional purgative should be taken, and if debility be produced by the bromides, wine and nourishing food, with cod-liver oil, strychnia, &c., should be used ; and the cold douche or shower-bath employed.

Nitrate of Lead in Sore Nipples.—In the Glasgow Medical Journal, J. G. Wilson, M. D. F. R. S. E., Professor of Midwifery in the Andersonian University, publishes the results of his experiments in the use of Nitrate of Lead in sore or excoriated nipples. He is satisfied of its superiority to any other agent which he has hitherto employed. He has found it to succeed when tannin, gallic acid, zinc, benzoin, borax, etc., failed to produce the desired effect. He recommends the following solution :

℞—Nitratis Plumbi, . . . gr. x.
Glycerine, . . . ℥ 1.

Solve. S. To be applied to the afflicted nipple after sucking. Care must be taken to wash the nipple previous to the next application of the infant. This, like all other applications, will sometimes fail, but it is certainly one of the best, and should not be forgotten.

Loss of Speech after Chloroform.—A servant girl, says the Alleg. Med. Centr. Quart., for the sake of the extraction of a tooth, inhaled chloroform for a short time. On awaking, she had lost the power of speech—could not utter any sound whatever, and remained in that state for five weeks in spite of various remedies—especially electricity. After this time she began to speak in a low tone, and was put under appropriate treatment. It is supposed that she suffered during anæsthesia, from rupture of some cerebral vessel.—*Detroit Jour. of Medicine and Pharmacy.*

PRESCRIPTION FOR SECONDARY AND TERTIARY SYPHILIS.—In the latter stages of Syphilis, the following formula is recommended :

℞—Potassi iodide, . . . . . . ʒ vij.

Bichloride hydrarg. . . . . . gr. ii. v.

Ext. Conii . . . . . . ʒ i

Syrup Stillingia, . . . . . . ʒ ii.

Syrup Sarp. Comp. . . . . . ℥ ii.

Tinct. Ci chonae, . . . . . ℥ iii. m.

S.—Teaspoonful three times daily.

It is well to give the dose with a teaspoonful of water, and one or two hours after meals.

CARBOLIC ACID IN GLEET.—Dr. T. J. Williamson, of Cincinnati, Ohio, in the Cincinnati Medical Repertory, states that he has been called upon to treat hundreds of cases of Gleet, and that he has never found any remedy half so efficacious as Carbolic Acid as follows :

℞—Glycerine, . . . . . . ℥ ss.

Carbolic Acid, . . . . . gr. viii.

M—Dip a No. 6 bougie in the above, and introduce up the al~~imentary~~ urinary canal, three times a day. The Doctor also recommends ʒ i. of Carbolic Acid to ℥ iv. of Glycerine, for Burns. We endorse his prescription.

BROMIDE OF LICHIUM IN EPILEPSY.—Dr. S. Weir Mitchell (in the Transactions of the College of Physicians, published in the American Journal of Medical Sciences, act 1870,) speaks favorably of the Bromide of Lithium in Epilepsy, in preference to the bromide of potassium. Several cases are given. He thinks the Bromide of Lithium, given in twenty-grain doses, three times a day, with the "Milk Cure," affords the best results in his hands, in the treatment of Epilepsy.

