# Errata

**Published:** 1871-02

**Authors:** 


### ERRATA.

In consequence of the hurry in getting up our first number, several typographical errors marred one or two of the formulas given. While such errors are of frequent occurrence in all Medical Journals, yet we assure our readers no pains shall be spared, on the part of the editors, to guard against any future mistakes of like character. Fortunately, the errors were not of sufficient gravity to have produced very unpleasant results.

Dr. Brown-Sequard's formula for epilepsy, on page 25, the infusæ columbæ, should read *six ounces* instead of six drachms. The proportions of the other articles are right.

On page 18, Dr. Broadwell's formula for Asthma should read: Ext. Stillingia comp. *one ounce*, instead of one drachm; Pottas. iodidi, *two and a half drachms*, instead of one and a half ounces. The other proportions and articles are right. We hope our friends will, with pencil or ink, correct these errors in their copies.

